# Comparison between the stochastic search variable selection and the least absolute shrinkage and selection operator for genome-wide association studies of rheumatoid arthritis

**DOI:** 10.1186/1753-6561-3-s7-s21

**Published:** 2009-12-15

**Authors:** Sudeep Srivastava, Liang Chen

**Affiliations:** 1 Molecular and Computational Biology, Department of Biological Sciences, University of Southern California, Los Angeles, California 90089, USA

## Abstract

**Background:**

Because multiple loci control complex diseases, there is great interest in testing markers simultaneously instead of one by one. In this paper, we applied two model selection algorithms: the stochastic search variable selection (SSVS) and the least absolute shrinkage and selection operator (LASSO) to two quantitative phenotypes related to rheumatoid arthritis (RA).

**Results:**

The Genetic Analysis Workshop 16 data includes 2,062 unrelated individuals and 545,080 single-nucleotide polymorphism markers from the Illumina 550 k chip. We performed our analyses on the cases as the quantitative phenotype data was not provided for the controls. The performance of the two algorithms was compared. Using sure independence screening as the prescreening procedure, both SSVS and LASSO give small models. No markers are identified in the human leukocyte antigen region of chromosome 6 that was shown to be associated with RA. SSVS and LASSO identify seven common loci, and some of them are on genes *LRRC8D*, *LRP1B*, and *COLEC12*. These genes have not been reported to be associated with RA. LASSO also identified a common locus on gene *KTCD21 *for the two phenotypes (marker rs230662 and rs483731, respectively).

**Conclusion:**

SSVS outperforms LASSO in simulation studies. Both SSVS and LASSO give small models on the RA data, however this depends on model parameters. We also demonstrate the ability of both LASSO and SSVS to handle more markers than the number of samples.

## Introduction

It is now feasible to perform large-scale, high-density genome-wide association studies (GWAS) to search for common genetic variants underlying common diseases (reviewed in [1,2]). Due to their computational feasibility, single-marker tests remain the primary tools in the analysis of GWAS data. However, most common diseases are complex and are caused by multiple genetic variants, each having only a small effect. The possible interactions among genetic variants and the interactions between genes and environment present additional challenges for disease mapping.

Because multiple loci contribute to complete diseases, testing markers simultaneously instead of one by one may increase statistical power. Advanced technology can provide us thousands of high-quality marker genotypes. To identify the correct set of genetic variants from thousands of markers, efficient and reasonable model selection algorithms are urgently needed. Two popular model selection methods have been proposed: the stochastic search variable selection (SSVS) [3] and the least absolute shrinkage and selection operator (LASSO) [4]. With SSVS, a latent variable *γ *is introduced to perform variable selection for regression models. If *γ*_*j *_= 1 the *j*^th ^variable is included in the model; if *γ*_*j *_= 0, the *j*^th ^variable is excluded from the model. A homogenous ergodic Markov chain can be generated by the Gibbs sampler. The empirical distribution of *γ *based on the Markov chain will converge to the actual posterior distribution of *γ *[5]. LASSO method proposed by Tibshirani is a shrinkage-based selection method for linear regression. LASSO minimizes the residual sum of squares subject to the constraint on the sum of absolute value of coefficients. This L1-Norm constraint produces shrunk coefficients with some of them exactly equal to zero, which leads to interpretable models. In 2004, Efron et al. proposed the least angle regression (LARS) [6], which is a computationally efficient model-selection algorithm. There is a close connection between LARS and LASSO. A simple modification of the LARS algorithm can yield all LASSO solutions. LASSO is one of the most popular model selection methods that involve minimization of the mean square error with respect to some constraints. On the other hand, SSVS is based on the Gibbs sampler, which belongs to the broader class of Markov-Chain Monte Carlo methods. Hence, a comparison of the two methods would be of great interest.

In this paper, we focus on two quantitative phenotypes related to rheumatoid arthritis (RA). The data was provided by the Genetic Analysis Workshop 16. RA is an autoimmune disease that causes chronic inflammation in joints, resulting in loss of function and disability. We applied SSVS and LASSO to the two quantitative phenotypes to identify genetic variants associated with RA. We compared the results based on 545,080 SNPs.

## Methods

### SSVS

SSVS uses a hierarchical Bayes model to identify associated variables [3]. Here we assume that the phenotype follows a multiple-regression model of a subset of the markers. The canonical regression setup is given by

where *Y *is *n *× 1, *X *= [*X*_1_,..., *X*_*p*_] is *n *× *p*, *β *= (*β*_1_,..., *β*_*p*_), and *θ*^2 ^is scalar.

A latent variable *γ*_*i *_is defined as the indicator whether marker *i *is selected in the model or not. SSVS uses the idea that the true posterior distribution of *γ*_*i *_values can be estimated by generating a Markov chain that converges to its stationary distribution. *β*_*i *_values are distributed according to a mixture distribution of two normal distributions with different variances, which enables the detection of associated variables:

The prior for *γ *is selected as a Bernoulli prior with the same parameter over all the *γ*_*i *_values:

Using simulation studies, we confirmed that changing (1/*p*) to other values such as 0.1,0.2,...,0.9 does not significantly change the power of SSVS (data not shown). The distribution of *θ*^2 ^conditional on *γ *is given by

which is equivalent to . SSVS implements the Gibbs sampler to generate the following Gibbs sequence using the conditional probabilities mentioned above:

which is an ergodic Markov chain that converges to its stationary distribution. We ran the Gibbs sampler for 2,000 iterations to achieve stationarity and then ran it for an additional 8,000 iterations to estimate the posterior probabilities of the *γ*_*i *_values.

### LASSO

LASSO tries to shrink the coefficients of independent variables and set most of the coefficients exactly equal to 0 to achieve a model with a small number of variables and a small mean square error. Using the same linear model as above,

where *Y *is the dependent variable, *X *are the independent variables, and *ε *is the independent error term, LASSO tries to minimize ||*Y *- *Xβ*||^2 ^subject to . Here *t *≥ 0 is the tuning or shrinkage parameter. An equivalent constraint would be

This is a quadratic programming problem but can be solved by a simple modification to the LARS algorithm by Efron et al. [6]. LARS, a modification of the classic model-selection method known as forward selection, orders regression covariates on the basis of their decreasing association with the output variable. By modifying LARS to enforce the sign consistency restriction of LASSO, we can generate all LASSO solutions corresponding to different values of the free parameter *λ*. Selecting the active model at a given iteration would give us LASSO solution corresponding to a particular value of *λ*. Hence, using a cutoff for the number of iterations allowed for the modified LARS, we can control *λ*.

### Simulation studies

For simulation studies, we considered 60 individuals. The genotype data was simulated as follows: we selected a set of markers from the Hapmap CEU population data. We utilized the Hapmap data because it provides enough markers at different marker densities. In the Hapmap CEU population data, there are a total of 60 independent individuals. For each sample, 60 markers were used for both SSVS and LASSO analysis. Among them, five markers were simulated to be associated with the phenotype (the causal markers). These causal markers were always included in the analysis. The phenotype was simulated from a linear model with the five causal markers:

where the *ε*_*i *_values are independent and identically distributed *N*(0,1). Different marker densities, coefficients (*β*), and SSVS parameters were considered. For each scenario, 1,000 simulations were performed. The average area under the curve (AUC) was calculated for each scenario. The AUC is the area under the receiver operator characteristic (ROC) curve, which is a plot of the sensitivity versus (1-specificity). An AUC of 0.5 represents a completely random guess. For SSVS, the AUC is calculated using the following formula modified from the method of Ma and Huang [8]:

where ***C ***is the set of indices of the causal markers and ***C***^*c *^is the set of indices of the non-causal markers. *n*_*c *_and  are the number of causal and non-causal markers respectively. For LASSO, the following formula is used to calculate the AUC:

where *λ*_*i *_is the iteration at which the *i*^th ^marker enters the model.

### Sure independence screening for the RA data

The genotype was coded as 0 for homozygous rare alleles, 1 for heterozygous alleles, and 2 for homozygous common alleles. Missing alleles were imputed according to the genotype frequency calculated from the available data for 0.7% of missing genotype data. As a prescreening step, markers with a minor allele frequency less than 0.01 were discarded. The phenotype data was log-transformed. To decrease the dimensionality of the marker data, sure independence screening (SIS) was performed [9]. SIS uses correlation learning to detect predictors in the true model. If *X *denotes the genotype data that are standardized columnwise, and *y *denotes the phenotype data, then *w *is defined as

SIS selects the largest component-wise magnitudes of the vector. Here, we selected the 1,000 markers with the largest correlations with the quantitative traits for SSVS and LASSO. Because the number of samples are 746 and 867 for the IgM and the anti-CCP phenotypes, respectively, we demonstrate the use of SSVS and LASSO to select a model in which the number of markers is greater than the number of samples. SIS reduces computational time required by the model-selection methods. SIS has been shown to retain important variables in the screened model with a large probability when a set of conditions are satisfied [9].

## Results

### Simulation studies

In the simulation studies, we considered different marker densities, causal marker effects, and SSVS parameters. We also compared the two methods with a standard single-marker F-test. The results are summarized in Table 1. SSVS consistently has a higher AUC value than LASSO and the single-marker test. We chose *σ *= 0.05 for SSVS and the values of *τ *were chosen according to the suggested ratios of  in George and McCulloch [3]. When the marker effect *β *is small (i.e., *β *= 0.5), LASSO performs worse than the single-marker F-test. In other scenarios, LASSO performs better than the single-marker F-test.

**Table 1:** Simulation results of QTL mapping

		AUC_b_				
		
Coefficient	Marker density^a^	SSVS (*τ *= 1)	SSVS (*τ *= 2)	SSVS (*τ *= 3)	LASSO	F-test
0.5	200	0.898	0.891	0.884	0.684	0.729
0.5	100	0.923	0.918	0.913	0.7	0.753
0.5	10	0.949	0.944	0.942	0.783	0.800
0.5	1	0.950	0.947	0.942	0.798	0.814
1	200	0.969	0.955	0.947	0.873	0.814
1	100	0.987	0.982	0.978	0.886	0.854
1	10	0.997	0.996	0.995	0.965	0.922
1	1	0.999	0.998	0.997	0.979	0.932
1.5	200	0.989	0.985	0.976	0.926	0.838
1.5	100	0.998	0.998	0.996	0.939	0.887
1.5	10	1	1	0.999	0.991	0.951
1.5	1	1	1	1	0.995	0.951
2	200	0.991	0.988	0.986	0.952	0.823
2	100	0.999	0.997	0.997	0.962	0.874
2	10	1	1	0.999	0.995	0.963
2	1	1	1	1	0.998	0.966

^a^measured in the average number of markers selected from every 1,000 markers of the Hapmap Phase I data

^b^The AUC values are the average values across 1,000 simulations. *σ *= 0.05

### RA

Markers with a minor allele frequency less than 0.01 were screened out. After this step, 425,555 markers for the IgM phenotype and 425,586 markers for the anti-CCP phenotype remained. After removing the samples with missing phenotypes, 746 samples for the IgM phenotype and 867 samples for the anti-CCP phenotype were left. The correlation between the phenotype and the genotype was calculated. The top 1,000 markers were chosen for each of the two phenotypes. Then, SSVS and LASSO were run on both phenotypes.

**Figure 1:**
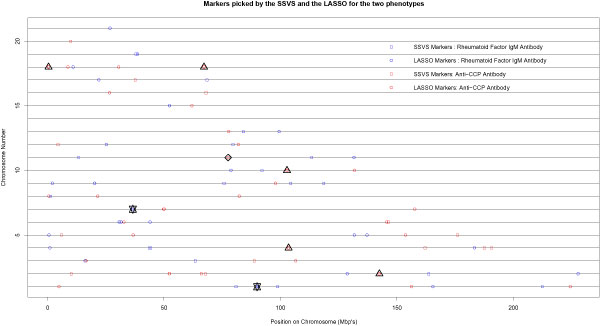
**Comparison of the outputs of SSVS and LASSO for the two phenotypes**. The squares show the markers selected by SSVS and the circles show the markers selected by LASSO. The stars enclose the loci identified by both SSVS and LASSO for the IgM phenotype. The triangles enclose loci identified by both SSVS and LASSO for the anti-CCP phenotype. The diamond encloses the locus identified by LASSO for the two phenotypes.

For SSVS, we used a cutoff of 0.9 for the posterior probabilities of markers being included in the model. The parameter values in the simulation studies, *σ *= 0.05 and *τ *= 1,2,3, were used such that  = 400, 1600, and 3600. For IgM phenotype, SSVS selected 20, 11, and 6 markers for the three  ratios. For the anti-CCP antibody phenotype, SSVS selected 18, 12, and 6 markers for the three  ratios. The positions of the markers selected by SSVS for *σ *= 0.05 and *τ *= 1 are shown in Figure 1. No common markers are identified for the two quantitative phenotypes.

The LASSO algorithm was run for 500 iterations unless it terminated earlier automatically because there were no more significant markers. The optimal cut-off for the number of iterations was selected using a 10-fold cross-validation. The minimum cross-validation error was achieved at 31 iterations for the IgM phenotype and at 33 iterations for the anti-CCP phenotype. It identified 31 significant markers for the IgM phenotype and 33 for the anti-CCP phenotype. The marker positions are also shown in Figure 1. No common markers were identified for the two phenotypes. However two markers, rs230662 for the IgM phenotype and rs483731 for the anti-CCP phenotype, are within 100 Kb of each other and both lie on the same gene *KTCD21 *at chromosome location 11q14.1. These markers have been enclosed in a diamond in Figure 1.

SSVS and LASSO identified a few common loci for the IgM phenotype and the anti-CCP phenotype. For the IgM phenotype, SSVS selected rs1938032 and LASSO selected rs12032393; both markers are located on the gene *LRRC8D *on chromosome 1 (enclosed by a star in Fig. 1). On chromosome 7, SSVS selected rs2392467 and LASSO selected rs13234380 for the IgM phenotype. Both markers are within 100 kb of each other and are shown in Figure 1 (enclosed by a star). For the anti-CCP phenotype, SSVS selected rs10183908 and LASSO selected rs972485 in the gene *LRP1B *on chromosome 2 (enclosed by a triangle). On chromosome 4, SSVS selected rs192068 and LASSO selected rs1540052, while on chromosome 10, SSVS selected rs807013 and LASSO selected rs17113682 (enclosed by triangles). Both marker pairs are within 100 kb of each other, but neither of them are located on annotated genes. SSVS and LASSO selected two common markers, rs2186830 and rs334438, on chromosome 18 (enclosed by triangles). Among them, marker rs2186830 is located on the gene *COLEC12*. None of these genes have been reported to be associated with RA in the literature.

## Discussion

SSVS and LASSO have advantages and disadvantages in their application to the model selection problem. SSVS is computationally intensive and cannot handle a very large set of markers. Hence, a more aggressive marker screening step needs to be done. The Gibbs sampler also requires the selection of prior parameters. In our simulation studies, by changing the prior of the *γ *values, we did not see a significant difference in the power of SSVS (data not shown). However, changing *σ *and *τ *will change the results. Further study is required on how to select these parameters. On the other hand, LASSO is very fast and hence the model is selected very quickly. It can handle large number of markers because the computational time in each step is proportional to the size of the model at that iteration. However, a big problem with LASSO is the selection of the cut-off. The Cp type score mentioned in Efron et al. [6] does not work in this data set because it does not give a small enough model (e.g., the model size using the Cp type score exceeds 200 markers for a given phenotype). LASSO may also overshrink parameters because it shrinks both non-significant coefficients and significant coefficients at the same rate. This can lead to overshrinking of the non-zero coefficients and result in models with large size as more variables are required to fit the data. To rectify this problem, cross validation is performed using the ordinary lease squares estimates of the model selected at each iteration rather than using LASSO estimates for *β *values. The problem of overshrinking can be minimized by using different penalties for different coefficients, which is performed in the adaptive LASSO [10]. In addition, it is very important to choose the prescreening steps carefully. A total of 1,000 markers were used for our studies to demonstrate the ability of SSVS and LASSO to handle more markers than the sample size. In future studies, it would be of great interest to address the number of markers chosen in the prescreening step to increase power.

For SSVS, we found that for different marker coeffcients (*β*), the best results were achieved by different values of *τ*. SSVS with *τ *= 1 had the maximum power compared with other values of *τ*. While for *β *= 2, SSVS with *τ *= 2 has the maximum power compared to *τ *= 1,3. It indicates that different values of hyper-parameters are required to detect markers with different effects.

SSVS and LASSO both rely on the assumption that the predictors are independent. However, the markers are dependent on each other due to linkage disequilibrium, which would need to be considered to make an accurate statistical inference. SIS also assumes independence, but is more robust to dependent markers than SSVS or LASSO. A modification of LASSO known as the adaptive LASSO [10] deals with correlated predictors by using adaptive weights for different predictors. It would be useful to compare the results of adaptive LASSO to that of LASSO.

SSVS and LASSO both assume a linear model and independent predictors. Using simulation studies, we have demonstrated that both SSVS and LASSO select common causal markers. When the markers are farther apart, i.e. they are independent of each other, and the prediction accuracy increases. In the real data, these assumptions may be violated. In complex diseases like RA, the individual marker effect is very weak and the marker effect may be nonlinear. The markers are not independent of each other due to high linkage disequilibrium. In addition, there are other confounding variables such as population structure and epistatic effects. All of the factors will affect the performance of the two methods to different degrees. Therefore, only a few common markers are selected by the two methods. It would be of great interest to study the behavior of SSVS and LASSO when these effects are incorporated into a more complex simulation model.

The human leukocyte antigen (HLA) region on chromosome 6 has been reported to be associated with the disease by various association and linkage studies [11,12]. However, we did not identify any markers within that region using LASSO or SSVS. This is an interesting result because markers in the HLA region correlate very strongly with the disease status. A possible explanation could be the missing quantitative phenotype data for the controls. QTL studies focus on quantitative traits instead of the binary disease status information. By incorporating disease status via a different method like logistic regression, random forests, and so on, one can build a more powerful model for the association study. This would be extremely useful in our data set because the disease status shows a very high association with the HLA region, but the quantitative phenotype data shows no association with the HLA region.

## Conclusion

In this paper, we compared SSVS and LASSO on simulated and real data. SSVS outperforms LASSO as well as the single-marker F-test in the simulation studies. The two methods were compared on the RA data provided by the Genetic Analysis Workshop 16 workshop after a dimension reduction using the SIS. Association studies were carried out for the two quantitative phenotypes using the two algorithms. The two methods identified two common markers for the anti-CCP antibody phenotype and two and three marker pairs that are located close to each other for the rheumatoid factor IgM phenotype and the anti-CCP antibody phenotype, respectively.

Some of these markers were found to be in annotated genes such as *LRRC8D*, *LRP1B*, and *COLEC12*. However these genes have not been reported to be associated with RA. In summary, SSVS and LASSO are very useful tools in GWAS studies for quantitative data.

## List of Abbreviations used

anti-CCP: Anti-cyclic citrullinated peptide; AUC: Area under the curve; GWAS: Genome-wide association study; HLA: Human leukocyte antigen; IgM: Immunoglobulin M; LARS: Least angle regression; LASSO: Least absolute shrinkage and selection operator; RA: Rheumatoid arthritis; ROC: Receiver operator characteristic; SIS: Sure independence screening; SSVS: Stochastic search variable selection.

## Competing interests

The authors declare that they have no competing interests.

## Authors' contributions

SS participated in the design of the study, performed the statistical analysis and helped to draft the manuscript. LC conceived of the study, and participated in its design and coordination and helped to draft the manuscript. All authors read and approved the final manuscript.
